# The pathologist is not a lonely sailor on the sea

**DOI:** 10.4103/2153-3539.77174

**Published:** 2011-02-26

**Authors:** Claudia Mello-Thoms

**Affiliations:** University of Pittsburgh, UPMC Shadyside Hospital, Cancer Pavilion, 5150 Center Avenue, Suite 301, Room 304, Pittsburgh, PA 15232

## SUMMARY

The advent of digital pathology and the consequent use of virtual microscopy have allowed insights into the processes used by pathologists to interpret a histopathological slide. Recently Roa-Peña, Gómez, and Romero[1] have added to the literature by studying the navigation strategies of four pathologists reading a very small case set of six virtual slides depicting different organ/disease combinations. The pathologists’ task was to identify the organ and, whenever possible, to provide a diagnosis of the disease process present. They used a custom-made interface, with two images depicted side-by-side in a single display: (1) a large thumb image showing the entire virtual slide, where all navigation, panning, and zooming activities were carried out by moving and/or modifying a small ‘view’ window; and (2) a large resolution image of the ‘view’ window. The authors reported that about half of the time spent reading the slide was used to closely examine areas of interest, whereas the other half was spent navigating through the piece, zooming, changing the ‘view’ window, etc. This is in agreement with a ‘search-and-focus’ strategy that has been reported elsewhere.[2] However, a unique aspect of this study is that the authors used different criteria to determine the pathologists’ ‘regions of interest’ (ROI). Among them (i) a group-based ‘coincidence’ criterion, according to which a given area had to be examined by more than one pathologist; and (ii) an ‘individual’ criterion, which took into account any areas that a single pathologist spent some time examining. Using these criteria, they reported a high ‘coincidence’ rate of areas visited by more than one pathologist, ranging from 41% in a gallbladder sample to 97% in a sample of the endomyometrium (mean: 70.5%). However, when the authors contrasted the ROIs that were determined using the ‘coincidence’ and the ‘individual’ criteria, their results suggested a low level of agreement. This led them to conclude that individual pathologists search each slide using their own unique strategies, and no general patterns can be observed despite the high ‘coincidence’ rates. While these results are somewhat surprising, before taking them at face value one needs to consider the very small sample size of the study and the custom-made interface with its own unique characteristics. I will address these results in the following commentary.

## COMMENTS

The ‘search-and-focus’ slide navigation strategy reported by Roa-Peña, Gómez, and Romero[1] has been previously observed by Krupinski *et al*.[2] and others.[3] There are two general types of scanning patterns: first, when many different areas of the slide attract visual attention for a short period of time and, second, when a selected number of areas are inspected for longer periods. In this dual strategy, areas that are examined for longer periods contain primarily diagnostic information, whereas areas that attract visual attention for shorter periods of time are auxiliary decision sites and may or may not contain relevant information. In Krupinski *et al*.[2] as in Roa-Peña, Gómez, and Romero[1] the ‘coincidence’ ROIs occurred much more frequently than the ‘individual’ ROIs. Furthermore, in Krupinski *et al*.[2] 80% of the areas examined by the pathologists were also commonly examined by other pathologists (that is, they were ‘coincidence’ ROIs), whereas in Roa-Peña, Gómez, and Romero[1] this ranged from 41%–97%. Perhaps this discrepancy was due to the fact that in Krupinski *et al*.[2] only one tissue type was used (breast core biopsies), whereas in Roa-Peña, Gómez, and Romero[1] four different histopathological specimens were present. This variety of tissue types could have influenced the pathologists’ navigation strategy (thus reducing the rates of agreement in the areas visited by multiple practitioners) by simply increasing uncertainty about what was being shown. The fact that the pathologists in Roa-Peña, Gómez, and Romero[1] spent 50% of their time interacting with the interface (i.e., ‘navigating’ the virtual slide) does suggest a greater need to physically explore the slide in order to determine tissue type and disease process. It also perhaps suggests navigation issues with the custom-made interface, but that is not easy to determine from the paper.[1]

Nonetheless, in clinical practice, when examining a slide pathologists know which histopathologic specimen they are looking at, which does suggest that a large percentage of ‘coincidence’ areas is to be expected if the case were to be reviewed by multiple practitioners. The importance of this finding is that it may serve as the basis upon which whole slide imaging may build a very successful future, because it may contain the key for automatic ROI determination and for the development of truly intelligent, integrative, computer-based diagnostic tools. For this to happen, future studies need to determine (i) which image-based ‘elements’ differentiate the ‘coincidence’ areas from others in the slide; (ii) how these ‘elements’ relate to higher level cognitive constructs used by pathologists when interpreting an image; and (iii) how to automatically mine these ‘elements’ from any given virtual slide. In this context, automated ROI determination can be accomplished by combining (i) and (iii), while integrative and useful computer aid can be obtained by determining (ii).

## References

[1] Roa-Peña L, F Gómez, E Romero (2010). An experimental study of pathologist’s navigation patterns in virtual microscopy. Diagn Pathol.

[2] Krupinski EA, Tillack AA, Richter L, Henderson JT, Bhattacharyya AK, Scott KM (2006). Eye-movement study and human performance using telepathology virtual slides. Implications for medical education and differences with experience. Hum Pathol.

[3] Tiersma ES, Peters AA, Mooij HA, Fleuren GJ (2003). Visualizing scanning patterns of pathologists in the grading of cervical intraepithelial neoplasia. J Clin Pathol.

